# Passive In-Line Chlorination for Drinking Water Disinfection: A Critical Review

**DOI:** 10.1021/acs.est.1c08580

**Published:** 2022-06-14

**Authors:** Megan Lindmark, Katya Cherukumilli, Yoshika S. Crider, Perrine Marcenac, Matthew Lozier, Lee Voth-Gaeddert, Daniele S. Lantagne, James R. Mihelcic, Qianjin Marina Zhang, Craig Just, Amy J. Pickering

**Affiliations:** † Department of Civil and Environmental Engineering, University of Iowa, Iowa City, Iowa 52242-1396, United States; ‡ Department of Civil and Environmental Engineering, University of California Berkeley, Berkeley, California 94720-2284, United States; § Energy & Resources Group, University of California Berkeley, Berkeley, California 94720-2284, United States; ∥ Division of Epidemiology & Biostatistics, University of California Berkeley, Berkeley, California 94720-2284, United States; ⊥ King Center on Global Development, Stanford University, Stanford, California 94305-2004, United States; # National Center for Emerging and Zoonotic Infectious Diseases, Centers for Disease Control and Prevention, Atlanta, Georgia 30329, United States; ∇ SAMRC/WITS Developmental Pathways for Health Research Unit, University of the Witwatersrand, Johannesburg, 2050, South Africa; ○ Tufts University School of Engineering, Medford, Massachusetts 02155-1012, United States; ◆ Department of Civil and Environmental Engineering, University of South Florida, Tampa, Florida 33620-5350, United States; ¶ Lichtenberger Engineering Library, University of Iowa, Iowa City, Iowa 52242-1396, United States; ● Blum Center for Developing Economies, University of California Berkeley, Berkeley, California 94720-2284, United States

**Keywords:** passive in-line chlorination, drinking water treatment, chlorine disinfection, resource-constrained settings, low- and middle-income countries, safely managed water supply

## Abstract

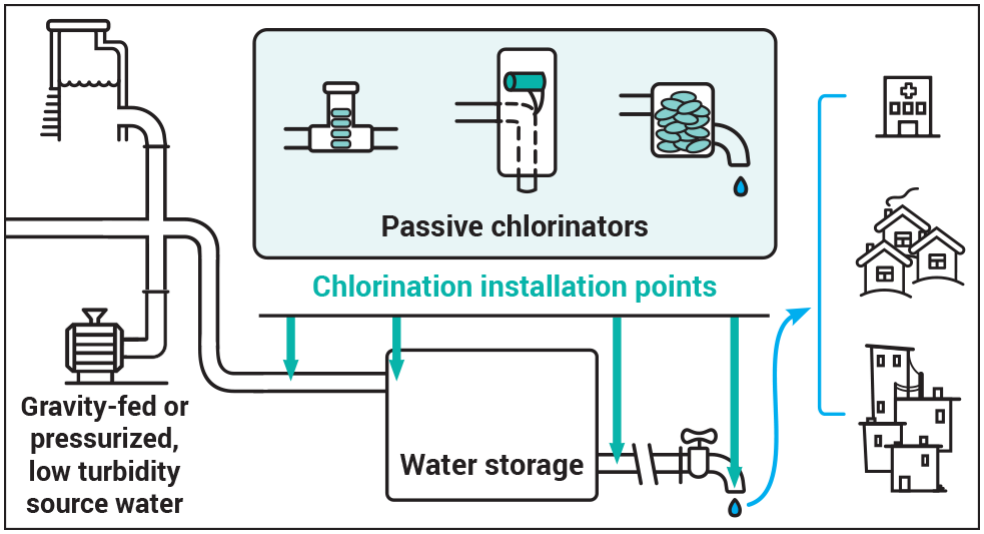

The world is not on track to meet Sustainable Development Goal 6.1 to provide universal access to safely managed drinking water by 2030. Removal of priority microbial contaminants by disinfection is one aspect of ensuring water is safely managed. Passive chlorination (also called in-line chlorination) represents one approach to disinfecting drinking water before or at the point of collection (POC), without requiring daily user input or electricity. In contrast to manual household chlorination methods typically implemented at the point of use (POU), passive chlorinators can reduce the user burden for chlorine dosing and enable treatment at scales ranging from communities to small municipalities. In this review, we synthesized evidence from 27 evaluations of passive chlorinators (in 19 articles, 3 NGO reports, and 5 theses) conducted across 16 countries in communities, schools, health care facilities, and refugee camps. Of the 27 passive chlorinators we identified, the majority (22/27) were solid tablet or granular chlorine dosers, and the remaining devices were liquid chlorine dosers. We identified the following research priorities to address existing barriers to scaled deployment of passive chlorinators: (i) strengthening local chlorine supply chains through decentralized liquid chlorine production, (ii) validating context-specific business models and financial sustainability, (iii) leveraging remote monitoring and sensing tools to monitor real-time chlorine levels and potential system failures, and (iv) designing handpump-compatible passive chlorinators to serve the many communities reliant on handpumps as a primary drinking water source. We also propose a set of reporting indicators for future studies to facilitate standardized evaluations of the technical performance and financial sustainability of passive chlorinators. In addition, we discuss the limitations of chlorine-based disinfection and recognize the importance of addressing chemical contamination in drinking water supplies. Passive chlorinators deployed and managed at-scale have the potential to elevate the quality of existing accessible and available water services to meet “safely managed” requirements.

## 1 Introduction

In 2015, the United Nations (UN) set Sustainable Development Goal (SDG) 6.1 to provide drinking water for all that is *safely managed*: available on premises, available when needed, and free of microbial and chemical contaminants.^1,2^ However, as of 2020, approximately 2 billion people—over 25% of the world’s population—still remain without access to safely managed drinking water.^3^ Conventional water treatment methods, including chlorination, filtration with biosand filters and ceramic pots, UV irradiation, and ozonation, can increase access to safely managed drinking water by inactivating or removing waterborne pathogens.^4^ Disinfection technologies can be applied to treat water sources at multiple institutional scales, including at the point of use (“POU”: household taps, stored water), at the point of collection (“POC”: community shared taps), and along municipal utility distribution systems. The success and scalability of disinfection technologies is dependent on numerous factors, including electricity access (for ozonation and UV irradiation),^5,6^ residual disinfection protection (only provided by chlorination), intermittency of water supply,^7^ user burden (especially for manual filtration and manual chlorination),^8^ local manufacturing and production capacity, and costs of the technology, installation, operation, and maintenance.

Chlorination has been widely used in resource-constrained settings because it is inexpensive, does not require electricity, and provides a free chlorine residual (FCR) to protect stored water from recontamination for a period of time.^9^ Although chlorination does not readily inactivate certain pathogens (e.g.,*Cryptosporidium*and*Giardia*)^10^ or remove chemical contaminants, it is highly effective at inactivating most microorganisms in water. Additional disadvantages of chlorine include the formation of disinfection byproducts (DBPs) and a taste or odor that can reduce user adoption rates. For these reasons, chlorination alone cannot guarantee safely managed drinking water, but there is substantial evidence that it can protect public health by reducing diarrheal risk and mortality.^11,12^ Previous reviews on drinking water chlorination have primarily addressed manual POU chlorination technologies applied in household^13^ settings and chlorine use in emergency response settings.^14,15^ While POU water treatment can be a strategic approach to control waterborne diseases in household or humanitarian settings with no alternative treatment options, numerous studies have highlighted the difficulty in achieving sustained effective use of POU products among low-income households.^16−18^

Passive chlorinators are defined here as devices that continuously and automatically dose chlorine prior to water collection without requiring active user input or electricity. It is critical to acknowledge that, although the chlorine dosing process is passive, passive chlorinators still require active operation and management efforts to refill chlorine, ensure that dosing accuracy is consistent, and guarantee that the treated water meets standards for adequate disinfection and end-user taste/odor preferences. Only one known review on gravity- and water-powered chlorinators was published in 2001.^19^ In the past two decades, many passive chlorinator devices have been developed and evaluated, presenting a need to consolidate this newly available evidence. The purpose of this critical review is to provide a detailed analysis of the available evidence on the demonstrated effectiveness of passive chlorinators and to identify specific contexts in which they have or have *not* been shown to elevate existing water supplies to the safely managed standard by providing an adequate dose of free residual chlorine. We analyzed peer-reviewed literature and non-peer-reviewed evaluations to (i) assess the performance and efficacy of various passive chlorinators to disinfect water in community and institutional settings, (ii) illustrate the advantages and limitations of current passive chlorinators, and (iii) identify future research areas and pathways to overcome existing barriers and improve water service delivery through passive chlorination. Furthermore, we provide context and guidance to researchers and practitioners who are developing, evaluating, and implementing passive chlorinators.

### 1.1 Methods for Reviewing Literature

A systematic literature search was conducted using search terms related to chlorine, drinking water treatment, and chlorination technologies on the PubMed, Proquest Dissertation and Theses Global, and Scopus databases (the detailed strategy is given in the Supporting Information). Only studies from the World Bank’s list of low- and middle-income countries (LMICs) were included. An initial screening of article titles was completed in Endnote by ML. On the basis of the title alone, articles on wastewater treatment or water treatment for industrial processes were excluded. Abstracts of the remaining articles were further screened for the same criteria, narrowing a list of 3671 articles down to 65 articles. Additionally, 13 relevant articles that were identified through reference tracing (citations in included articles) or that were explicitly known to coauthors were added, resulting in a total of 78 full-text articles reviewed by our team.

The following criteria were used to define passive chlorinators and determine which papers should be included in this review: (i) devices have a demonstrated application for drinking water disinfection at the multihousehold scale in LMICs, (ii) devices do not require electricity for operation, and (iii) devices are capable of automatically (i.e., passively) and continuously dosing chlorine.

Fifty-one articles were excluded because the technologies presented did not meet this definition of passive chlorinators. Two graduate theses were excluded because they reported the same data published in other included papers. All chlorinators included and excluded were double-checked by at least one additional author, and if there was uncertainty, a third individual resolved the discrepancy. Ultimately, 19 peer-reviewed articles, 3 NGO reports, and 5 graduate theses were selected for analysis and discussion in this review. To ensure the inclusion of passive chlorinators used in field settings but not discussed in peer-reviewed literature, an anonymous Google Form survey was sent to nongovernmental organizations (NGOs) and researchers within the authors’ networks (see the Supporting Information). This survey included questions about existing passive chlorinator implementations, including geographic locations, settings, and ongoing monitoring and evaluation efforts. The 10 survey responses received described implementations of 13 different chlorinators, 7 of which were not formally evaluated otherwise. Survey respondents either provided links to publicly available evaluation reports or shared internal reports for inclusion in this review.

Authors aggregated the following information from selected papers and survey responses: device name, product availability, power requirements, chlorine source, mechanism of chlorine dosing and control, implementation location and scale, system type and location (within the distribution system), water source and flow regime compatibility, population served, and associated costs (device, installation, and chlorine refills). Additional data measuring technical performance were collected, including free chlorine residual (FCR) (mean, standard deviation, range, proportion of samples in specified range) and fecal indicator bacteria (FIB) (log values, log reduction, concentration, proportion of samples in specified range).

### 1.2 Passive Chlorinator Search Results

The passive chlorinators discussed in this review have been implemented for drinking water disinfection in resource-constrained settings using a variety of chlorine sources and dosing control mechanisms (Table 1). Table 1 presents the 27 chlorinators found in this review process, along with information on device commercial availability or “constructability”, chlorine dosing and control mechanism, reported flow regime and system compatibility, and associated costs. Overall, 81% (22/27) of the reviewed chlorinators were erosion or dissolution-based, being reliant on water flow through the device to dissolve solid chlorine granules or tablets. A total of 90% (20/22) of erosion chlorinators included here (and 74% (20/27) of all passive chlorinators included) used tablets rather than granular chlorine. The remaining 19% (5/27) of passive chlorinators used liquid chlorine dosed via the Venturi effect, pressure differentials, or suction.

**Table 1:** Characteristics of Passive Chlorinators Identified in Literature Review and NGO Survey^a^

passive chlorinator^c^ (product info)	chlorine dosing mechanism	flow regime	current system compatibility	dosing control mechanism	associated costs (USD, inflation adjusted)
Tablet					
ADEC Clorador/Adapted CTI-8***^b^ (self-constructed)	dissolution	gravity or pressurized	prior to storage tank	manual bypass valve	device: $150–200.00
					chlorine refill: 10 tablets included in device cost^24^
A’jin Chlorinator*** (not reported)	dissolution	not reported (evaluation underway)	not reported (evaluation underway)	not reported (evaluation underway)	device: cost not reported, evaluation underway
AkvoTur (self-constructed)	dissolution	gravity	after storage tank, pretap	number of slits in the tablet chamber exposed	device: $7.00^21^
Arch Chemical Pulsar 1 (commercially available)	dissolution + Venturi	gravity or pressurized	not reported	manual bypass valve + internal slit position	no costs reported
Aquatabs Flo (commercially available)	dissolution	gravity	prior to storage tank	screw restricting outflow	device: $20.00^21^ (including tablets)–$46.00^17^ (cost of full installation with additional hardware)
Aquatabs Inline*** (commercially available)	dissolution	gravity or pressurized	prior to storage tank or tap	manual bypass valve	device: $58.00^25^
Aquaward (commercially available)	dissolution	gravity or pressurized	prior to storage tank	manual bypass valve	device: $608.35^26^
chlorine dosing bucket (self-constructed)	dissolution	gravity	at the tap	manual bypass valve	device: $50.00^21^
CTI-8 (self-constructed)	dissolution	gravity or pressurized	prior to storage tank	manual bypass valve	device: $267.06^27^
					device: $49.50^25^
floating chlorinator (not reported)	dissolution	N/A	floating in well or storage tank	no. of tablets, slit position	device: $7.00^21^
Fluidtrol Process Technologies Chlorinator (research grade)	dissolution	pressurized	prior to full distribution system	not reported	no costs reported
MINSA (Panama) chlorinator (self-constructed)	dissolution	gravity or pressurized	prior to storage tank	none	device: $32.95^28^
					device: $15.00^21^
Norweco (commercially available)	dissolution	gravity	prior to storage tank	manual bypass valve	eevice: $82.50^25^
PurAll 50H (commercially available)	dissolution	gravity	handpumps	none	eevice: $60.00^25^
PurAll 100 (commercially available)	dissolution	gravity or pressurized	prior to storage tank or tap	manual bypass valve	device: $662.00^17^
					installation: Cost of full installation + installation hardware included
T-shaped erosion chlorinator (self-constructed)	dissolution	gravity or pressurized	prior to storage tank	manual bypass valve	no costs reported
Waterway + OceanBlue (commercially available)	dissolution	gravity	prior to storage tank	none	device: $168.24^29^
Water Mission Erosion Chlorinator*** (commercially available)	dissolution	gravity or pressurized	not reported	linear flow control valve	no costs reported
Vulcano Code 102200*** (commercially available)	dissolution	gravity or pressurized	prior to storage tank	manual bypass valve	no costs reported
Water4 NuPump*** (not reported)	dissolution	not reported	not reported	not reported	no costs reported
Liquid					
AguaClara (research grade)	linear chemical dose controller^30^	gravity	multi-stage water treatment plant	linear chemical dose controller^30^	device: $49063.00–83382.00^31^ (cost of full treatment plant, not just chlorinator)
Blue Tap (research grade)	Venturi + patented hydraulic control	gravity or pressurized	prior to storage tank	needle valve regulator	device: $160
Nirapad Pani (research grade)	suction	pressurized	handpump (inlet)	internal regulator	device: $26.12^32^
Stanford-MSR Venturi (research grade)	Venturi	gravity or pressurized	at tap	needle valve regulator	device: $34.00^25^ (estimated cost at scale)
Zimba (commercially available)	suction	gravity	handpump	not reported	device: $112.16^33^
Granular					
hypochlorinator (self-constructed)	dissolution	gravity or pressurized	prior to storage tank	manual bypass valve	no costs reported
pot chlorinator (self-constructed)	dissolution	N/A	floating in well or storage tank	none	device: $3.12^34^

a An unnamed chlorinator evaluated by Ali et al.^35^ was not included in the table because the device name was not reported or known by the original authors. The evaluation of this device is summarized in Table 2.

b Chlorinators reported via a practitioner survey are indicated with asterisks (***).

c Research grade specifically denotes a device that has only been designed or utilized within a research context. These devices are therefore not currently commercially available.

Many devices (41%, 11/27) used a manual valve and a bypass placed in parallel with the chlorinator to adjust the flow rate of the untreated water and ensure appropriate chlorine dosing. Alternatively, a few of the passive chlorinators (19%, 5/27) used internal valves (e.g., needle valves or linear control valves), which could be adjusted to increase or decrease the chlorine dose independent of flow rate. In the place of valve(s), at least three chlorinators had a series of slits holding tablets to control the influx of water into the chamber, which could be rotated to expose fewer or more of the slits to adjust dosing. For some devices where the chlorine dose was automatically proportional to the flow rate (e.g., Venturi), internal valves offered an additional way to control dosing more precisely. For 26% (7/27) of chlorinators, a dosing control mechanism was not reported or was not present. Devices with a mechanism to adjust chlorine dosing (independent of flow rate) enable operators to account for variable source water chlorine demand and maintain sufficient FCR. Depending on the source water turbidity and chlorine demand, pretreatment steps may be necessary to ensure consistent dosing. The AguaClara treatment system incorporated coagulation, flocculation, and settling tanks to account for the variable turbidity of incoming source water.^20^ Several other evaluations reviewed here coupled chlorinators with other forms of treatment such as gravity-driven membrane filtration systems^21^ or natural (gravel and sand) filtration upstream of chlorination.^22,23^

The capital cost of commercially available passive chlorinators ranged from $3 to $662, with an average cost of $140 (values reported in 2021 USD, adjusted for inflation). This range does not include the AguaClara price point of $49063, which is the cost of the full water treatment system and not just the chlorinator. Dossegger et al.^21^ accounted for maintenance expenses, labor, and chlorine refills in their cost estimates of water treated by passive chlorinators ($0.01–$1.07 per 1000 L) versus a manual chlorine dispenser ($0.18–$0.99 per 1000 L). The estimated operating cost of 4 out of 6 passive chlorinators in this study (i.e., floating chlorinators, chlorine-dosing bucket, T-Chlorinator, and Akvotur) was lower than $0.10/1000 L, allowing them to be considered as economically viable. Crider et al.^17^ evaluated the cost of implementing, refilling, and monitoring two passive chlorinators (PurAll 100 and Aquatabs Flo) in rural communities in Nepal. All devices and refills were purchased locally at market prices. On consideration of the cost of the device and initial installation, including all site-specific hardware and fittings, the PurAll 100 was significantly more expensive ($662.00) than the Aquatabs Flo ($46.00). However, the cost of chlorine per cubic meter (1000 L) of treated water was $0.06 for PurAll and $0.09 for Aquatabs Flo, calculated on the basis of the cost of chlorine cartridge refills and the average water volume treated per refill. The site-specific refill costs ($0.06–$0.09 per 1000 L) and costs for monitoring ($0.05–0.07 per 1000 L) were similar for the Aquatabs Flo and the PurAll.

## 2 Implementation and Evaluation Studies

In this section, we summarize evaluation findings, settings where chlorinators have been implemented, and generalizable insights for future research and implementations. We identified 27 studies published between 2001 and 2021 that evaluated passive chlorinators (Table 2). Effectiveness metrics include FCR and FIB measurements. Water samples were most commonly collected from the POC: for example, a shared tap stand or tap connected to a storage tank. In some cases, water samples were collected at the POU, at a point upstream of the POC such as at the point of treatment (for system-level treatment), or from a storage tank post treatment.

**Table 2:** Evaluations Conducted on Passive Chlorinators Identified in Peer-Reviewed Literature

						effectiveness metrics^a^^–^d	
chlorinator	evaluation reference	country	implementation scale	system type and location (within distribution or water delivery network)	population served	FCR(mg/L) mean (SD) [range] (%) sample target	FIB log reduction (%) sample target
Tablet							
AkvoTur	Dossegger et al.^21^	Uganda	communities	gravity-driven membrane filtration kiosks	not reported	2.1 (0.5) [0.8–3.6]	not reported
						67% @ 1.5–2.5	
Arch Chemical Pulsar 1	Fitzpatrick^36^	Ghana	lab and noncommunity field site	N/A	N/A	[0.5–7.0]	not reported
						With system modifications	
Aquatabs Flo	Dossegger et al.^21^	Uganda	communities	gravity-driven membrane filtration kiosks	not reported	1.1 (0.6) [1.7–3.6]	not reported
						57% @ 1.5–2.5	
	Voth-Gaeddert and Schrank^37^	N/A	N/A	lab (flow rates 2–21 Lpm; modification for POU/POC)	N/A	Normal: [1.5 to >3.5]	not reported
						@ 2 Lpm 2.1	
						@ 10 Lpm 2.3	
						@ 18 Lpm 3.5	
						modified for POU/POC: [0.3–2.3]	
						@ 2 Lpm 0.5	
						@ 10 Lpm 0.3	
						@ 18 Lpm 1.2	
	Pickering et al.^38^	Bangladesh	Urban compounds	Shared taps served via municipal piped supply	50 communal water points	0.33 (0.28)	0.84 log *E. coli* reduction in treated water compared to control
						80% >0.1	
							control: 5.49 CFU/100 mL
							treatment: 0.8 CFU/100 mL
							no E. coli detected in 85% of samples
	Marcenac et al.^39^	Tanzania	rural health care facilities	Rainwater harvest tank taps, standpipe taps, and elevated storage tank inlets	9 healthcare Facilities	rainwater harvest tank tap (*n* = 8): 0.93 (0.57) [0–3.4]	not reported
						standpipe tap (*n* = 1): 0.35 (0.13) [0.2–0.9]	
						elevated tank inlet (*n* = 3):0.29 (0.25) [0–1.2]	
	Crider et al.^17^	Nepal	rural community	at inlet of storage tank, predistribution network	28 households	74–86% > 0.1	1.02 log CFU *E. coli* reduction from upstream to post-treatment taps at endline, on average
							upstream: 0.83 log CFU/100 mL
	Smith et al.^40^	Bangladesh	low-income informal housing settlements + middle-income apartments	at inlet of storage tank, predistribution network or tap	∼65 landlords and respective housing units	0.42 (0.48)	not reported
						89% ≥0.1	
Aquaward	Brignoni^26^	Puerto Rico	rural community	at inlet of storage tank, predistribution network	1000 people	1.06 [0.5–1.7]	total coliform % reduction: [75–100%]
							initial total coliform concentration, pretreatment: 0–9000 cfu/100 mL
							final total coliform concentration, post-treatment: 0–20 cfu/1000 mL
							fecal coliform % reduction: [82–100%]
							initial fecal coliform concentration, pretreatment: 0–110 cfu/100 mL
							final fecal coliform concentration, post-treatment: 0–20 cfu/1000 mL
chlorine dosing bucket	Dossegger et al.^21^	Uganda	communities	gravity-driven membrane filtration kiosks	not reported	1.7 (0.9) [.3–3.5]	not reported
						40% @1.5–2.5	
CTI-8	Taflin^27^	Nicaragua + Guatemala	rural communities	at inlet of storage tank, predistribution network	32 communities	not reported	not reported
	EOS, Ministry of Health report^22^ ***	Nicaragua	rural communities	at inlet of storage tank, predistribution network	21 communities	not reported	not reported
	CTI report^23^ ***	Nicaragua	rural communities	at inlet of storage tank, predistribution network	70 communities	not reported	not reported
floating chlorinator	Dossegger et al.^21^	Uganda	communities	gravity-driven membrane filtration kiosks	not reported	1.5 (0.9) [0.1–3.1]	not reported
						37% @ 1.5–2.5	
	Garandeau et al.^41^	Liberia	internally displaced persons camp	floating in well	not reported	[0.2–1.0] (modified floating chlorinator)	not reported
Fluidtrol Process Technologies Chlorinator	Martin^42^	Haiti	large community	predistribution network (not specified)	3000	69% >0.5	not reported
MINSA (Panama) Chlorinator (“T-Chlorinator”)	Orner et al.^28^	Panama	rural indigenous community	at inlet of storage tank, predistribution network	325 people	1 tablet: [0.02–0.24]**	not reported
						3 tablets: [0.02–0.44]	
	Yoakum^43^	Panama	rural indigenous community	at inlet of storage tank, predistribution network	183 people	2 tablets: [0.02–0.2]**	not reported
						3 tablets: [0.27–0.63]	
	Dossegger et al.^21^	Uganda	communities	gravity-driven membrane filtration kiosks	not reported	2.0 (0.3) [1.1–3]	not reported
						90% @ 1.5–2.5	
Norweco	Rayner et al.^44^	Haiti	natural disaster/complex emergency, community setting	at inlet of storage tank, predistribution network	not reported	0% detectable chlorine	28% <1 CFU *E. coli*
							47% 1–10 CFU *E. coli*
							23% 11–100 CFU *E. coli*
							2% 101–1000 CFU *E. coli*
PurAll 50H	Sikder et al.^45^	Cox’s Bazaar, Bangladesh	2 refugee camps	shared handpump	44000	0.9 (1.3)	89% <10 CFU/100 mL
						44% > 0.2	
PurAll 100	Crider et al.^17^	Nepal	rural community	at inlet of storage tank, predistribution network	27 households	90–100% >0.1	1.32 log CFU *E. coli* reduction from upstream to post-treatment taps at endline, on average
							upstream: 1.02 log CFU/100 mL
T-Shaped Erosion Chlorinator	Henderson et al.^46^	Honduras	rural communities	at inlet of storage tank, predistribution network	5 communities	1.2 (0.06) **	not reported
						90.3% >0.2	
Waterway + OceanBlue^c^	Blair et al.^29^	Dominican Republic	rural community	in-line prestorage tank	not reported	[0.05–1.74]	not reported
	Ngo et al.^47^	Dominican Republic	rural community	in-line prestorage tank	not reported	[0.62–1.89] *	not reported
Liquid							
AguaClara	Brooks et al.^20^	Honduras	large communities (5)	full scale water treatment plant	11400 (total)	5 separate AguaClara systems:*	5 separate AguaClara systems:*
						0.9	*E. coli* Reduction:
						0.14	>99.5%
						0.01 (limit of detection, system was not chlorinating during study period)	>99.0%
							>97.6%
							>97.8%
							>91.5%
						0.27	initial *E. coli* concentration, pretreatment range: <10 to >100 mpn/100 mL
						0.2	
							final *E. coli* concentration, post-treatment in all 5 systems: no detectable *E. coli* (<0.5 mpn/100 mL)
Nirapad Pani	Pickering et al.^32^	Bangladesh	urban compounds	at inlet of storage tank	10 compounds (average 19 households/compound)	0.66 (0.57)	78% <1 CFU *E. coli*
						80% >0.2	
Stanford-MSR Venturi^b^	Powers et al.^48^	Kenya	rural and urban communities	at the tap of community water kiosks	not reported	0.55 (0.29)	not reported
						[0.0–1.59]	
						88% >0.2	
						86.2% @ 0.2–1.2	
Zimba	Amin et al.^33^	Bangladesh	neighborhoods (6)	shared handpumps	not reported	1.3 (0.54)	0.43 log CFU *E. coli* reduction in treated water compared to control
						100% @ 0.2–2.0	
							Control: 3.47 CFU/100 mL
							Treatment: 1.29 CFU/100 mL
							72% < 1 CFU *E. coli*
un-named liquid chlorinator	Ali et al.^31^	South Sudan	refugee camps (3)	in-line, pre storage tank	camp 1: 15500	camp 1: 0.9 (1.2) [0.01–4.60]	not reported
					camp 2: 37200	camp 2: 1.2 (0.3) [0.6–2.3]	
					camp 3: 15800	camp 3: 1.4 (1.2) [0.1–5.2]	
Granular							
hypochlorinator	Henderson et al.^46^	Honduras	rural communities	at inlet of storage tank, predistribution network	8 communities	0.67 (0.50)	not reported
pot chlorinator	Cavallero et al.^34^	Guinea Bissau	cholera outbreak, community	suspended in well	6 neighborhoods	24 h post-installation: 62% @ 0.2–5.0	not reported
						48 h: 15% @ 0.2–5.0	
						72 h: 4% @ 0.2–5.0	
	Garandeau et al.^41^	Liberia	internally displaced persons camp	suspended in well	not reported	[0–10]	not reported

a Free chlorine values are typically reported in the following format: mean (sd) [range], % at target value, in mg/L. *E. coli*measurements are typically reported in the following format: log reduction, % within target range cfu/100 mL or [range of % reduction]. Log reduction is calculated as follows: log reduction = log_10_(initial concentration of bacteria/final concentration of bacteria). % reduction is calculated as follows: % reduction = (initial concentration of bacteria-final concentration of bacteria) × 100/initial concentration of bacteria.

b Testing of an early Stanford-MSR Venturi prototype doser in Dossegger et al.^21^ was not included in this table. Evidence from the paper suggests that it was installed incorrectly with operational flows below the intended flow rates.

c Blair et al.^29^ and Ngo et al.^47^ evaluated two types of chlorinators, but the results were reported in aggregate for both chlorinators.

d Evaluations where water samples for FCR and*E. coli* were not from the POC are denoted by *(POU samples) and **(storage tank samples, upstream of POC).

### 2.1 Technical Performance Evaluations

Studies assessed the technical efficacy of passive chlorinators directly (by measuring FCR at multiple time points) and/or indirectly (by using*E. coli* measurements as an indicator for disinfection). Although WHO guidelines for residual in piped water systems recommend 0.2–0.5 mg/L FCR as adequate for disinfection,^49^ some studies defined adequate chlorine delivery as any amount of measurable free chlorine above the limit of detection^17,32,38,40,44,45^ or used their own range of desired doses on the basis of other standards.^21,33,34,42,46,48^ Dosing consistency was reported as the percentage of samples with chlorine concentrations within the study’s indicated range (Table 2).

#### 2.1.1 Comparison of Passive and Alternative Chlorination Methods

Evaluations comparing passive chlorinators with alternative water chlorination strategies suggest that, in most implementation settings, passive chlorinators outperform manual and household chlorination methods on the basis of dosing consistency and mean FCR, and also often due to low sustained usage of manual options.^21,32^ A small randomized controlled trial by Pickering et al.^32^ demonstrated that manual household chlorination underperformed in comparison to a passive chlorinator (Nirapad Pani), which consistently dosed adequate chlorine in households over longer time periods (i.e., 80% of the time) because of diminishing adherence to household chlorination after promotion visits concluded. Similarly, Dossegger et al.^21^ demonstrated that the adherence to manual doser use was extremely variable, ranging from 5% to 87%, although the manual doser in their study performed as good or better than five (out of six) passive chlorinators (Table 2).^21^

Another study by Sikder et al.^45^ compared a handpump passive chlorinator (PurAll 50H) to centralized piped water chlorination and batch-level bucket chlorination. All households (100%, *n* = 159) with water treated by large-scale piped water chlorination had “low risk”^49^ water (<10*E. coli* CFU/100 mL), followed by passive chlorination (89%, *n* = 180 households) and batch-level bucket chlorination (71%, *n* = 148 households). However, passive chlorination had the lowest percentage of households served with an adequate chlorine residual on the basis of the WHO infrastructure guideline (0.2–0.5 mg/L FCR).^45^ Although this difference may be a result of the implementation setting (see section 2.3), the authors ultimately did not recommend use of the PurAll 50H passive chlorinator because the chlorine dose could not be adjusted.^45^

#### 2.1.2 Dosing Mechanisms and Device Performance

On average, the passive chlorinators reviewed here delivered average FCR concentrations ranging from 0.14 to 1.7 mg/L and had dosing consistencies (i.e., percent collected samples with target FCRs greater than 0.1 mg/L) ranging from 37% to 100% (Table 2). Evaluations^17,33,38^ measuring*E. coli* at the POC or POU found that passive chlorination resulted in 0.43–1.3 log reduction of*E. coli* (i.e., 62.8–95%). However, not all studies (other than those given in Table 2) reported initial*E. coli* concentration, limiting an interpretation of the removal efficiencies. Additionally, other researchers reported the number of water samples at or below certain*E. coli* concentrations or, in some cases, the concentration or inactivation of fecal and total coliforms. This variability in reporting metrics for bacterial contamination reduction makes it difficult to compare these chlorinators’ effectiveness and performance.^26,32,44,45^

Chlorine dosing consistency and accuracy were also variable on the basis of the type of passive chlorinator and primary mechanism used to introduce chlorine to the water. Solid tablet chlorinators make up the largest proportion of passive chlorinators reviewed here, but they also represent the most diverse group in terms of effectiveness, with dosing consistencies ranging from 40% to 90% (Table 2). Solid tablet chlorinators, which are variations of a T-shaped chlorinator or consist of a container holding chlorine tablets, can be installed in-line or at the end-of-line (i.e., at the POC or at the inlet for a storage tank). These devices can either be constructed out of locally available materials in many settings (ADEC, CTI-8, A’jin, MINSA, T-shaped erosion chlorinator, AkvoTur, chlorine dosing bucket) or purchased commercially (Aquatabs Inline, Aquatabs Flo, Aquaward, PurAll 100, Waterway, Ocean Blue, Vulcano Code 102200).

Dossegger et al.^21^ evaluated the performance of many of these passive chlorinators on the basis of their dosing consistency. The self-constructed T-Chlorinator performed most effectively (90% samples maintained between 1.5 and 2.5 mg/L FCR), followed by Akvotur (67%), Aquatabs Flo (57%), chlorine dosing bucket (37%), and floating chlorinator (37%). An additional non-peer-reviewed report produced by the NGO Evidence Action compared six passive chlorinators including CTI-8, Norweco LF1000, Aquatabs Inline, Aquatabs Flo, Stanford-MSR Venturi, and PurAll 50H.^25^ While they did not report dosing levels or directly compare devices under the same flow rate conditions, they reported choosing to pilot the Norweco LF1000 and the CTI-8 chlorinators in Kenya because they performed well in laboratory tests, were commercially available (or could be built with local materials), and were compatible with storage tanks in their program area.^25^ Crider et al.^17^ evaluated the Aquatabs Flo and PurAll 100 chlorinators over approximately 1 year in rural communities in Nepal with piped gravity-fed water supplies. The dosing consistency (i.e., percent of collected samples with FCR >0.1 mg/L) of PurAll 100 (90–100%) was notably higher than that of the Aquatabs Flo (74–86%).^17^ All samples from the midline and end-of-study assessments showed a reduction in*E. coli* from the source to the tap, even as the source water quality worsened. Although both devices were found to effectively improve water quality in community piped networks with shared taps, recontamination was a problem. Post-collection FCR decreased, and in some cases,*E. coli* levels in household-stored water exceeded those in prechlorination samples.

Inconsistent chlorine dosing was most commonly observed in chlorine dosing buckets, hypochlorinators, and floating and pot-style passive chlorinators. These devices use a tablet or granular chlorine that rapidly dissolves due to inundation to produce a concentrated chlorine solution that dissipates into the water.^21,34,41,46^ An evaluation conducted across rural communities in Honduras demonstrated that a passive chlorinator slowly dissolving calcium hypochlorite tablets^46^ directly into the influent water was far more effective (mean FCR: 1.2 mg/L) than a hypochlorinator (mean FCR: 0.67 mg/L), which relied on rapidly mixing a stock solution made from granular calcium hypochlorite with unchlorinated influent water flowing into the tank through a bypass.

Passive chlorinators reliant on liquid chlorine (e.g., Stanford-MSR Venturi, Nirapad Pani, and Zimba) provided consistent and adequate FCR dosing across different implementation settings, with Zimba (80%) and Venturi (97%) having the highest dosing consistencies (Table 2).^32,33,48^ Stanford-MSR Venturi relies on the Venturi effect to pull chlorine into running water flowing through the chlorinator.^48^ Zimba is a batch doser that relies on water reaching a specified level to trigger the addition of a fixed amount of chlorine.^33^ Nirapad Pani, which was designed for use with handpumps (but not commercialized), uses suction generated by operating the handpump to pull liquid chlorine into the water.^33^ These evaluations suggested that, unlike the solid tablet chlorinators, some liquid-dosing chlorinators can be installed directly at the tap and still achieve consistent dosing. It is difficult to make a generalized comparison of the performance of solid versus liquid dosers, as there are far fewer evaluations of the limited liquid chlorinators available in the market.

#### 2.1.3 Impact of Device Installation and Positioning

The technical performance of passive chlorinators is also dependent on proper installation and positioning of the device within a water delivery system. Voth-Gaeddert and Schranck^37^ conducted performance evaluations of Aquatabs Flo to assess how changes in flow rate (2–18 Lpm) and installation alignments (i.e., tilting the device forward or sideways) affected FCR values under controlled conditions. Researchers reported that device misalignments negatively influenced the capacity of the Aquatabs Flo to dose adequate chlorine, necessitating careful installation for effective disinfection. In addition, the researchers successfully modified the chlorinator to maintain FCR in the necessary range by partially blocking entry to the tablet compartment using readily available plastic tubing.^37^ Martin^42^ conducted laboratory-scale experiments to optimize the design of a large-scale tablet chlorinator and to parametrize fluid dynamic modeling, prior to implementation in Cange, Haiti. Through laboratory experiments, the inlet and outlet positions of the final chlorinator were designed so that, in 69% of post-installation samples, chlorine levels were maintained above 0.5 mg/L.^42^

In addition to appropriate installation, proper device operation and maintenance practices also likely explain significant differences in effectiveness across different implementation settings. For example, the MINSA tablet chlorinator installed in Panama by Orner et al.^28^ achieved a much lower range of chlorine concentrations (0.02–0.44 mg/L) in comparison to the installation in Uganda by Dossegger et al. (1.7–2.3 mg/L).^21^

### 2.2 Health Impact Evaluations

Although many studies included here measured*E. coli* concentrations in water, a widely used indicator for waterborne disease risk,^50,51^ the presence of*E. coli* alone does not always correlate with the presence of other waterborne pathogens.^52^ Similarly, post-treatment FCR measurements do not fully capture the expected public health benefits of improved microbiological water quality.^4^ The only peer-reviewed evaluation that measured health outcomes of passive chlorination was a blinded, randomized placebo-controlled trial conducted by Pickering et al.^38^ in urban Bangladesh. The researchers tested the effect of implementing the Aquatabs Flo passive chlorinator on the reported incidence of diarrheal disease in children under five. During the 14-month study period, children who received drinking water treated by the passive chlorinator had significantly lower diarrhea prevalence (23% reduction) in comparison to the control group, which received water dosed with vitamin C. A total of 80% of the water samples collected at the treatment group’s taps had detectable (>0.1 mg/L) FCR, as opposed to the control group water samples (0%). Two non-peer-reviewed evaluations conducted by Compatible Technology International (CTI) in 2015 and EOS International in 2018 assessed the impact of the CTI-8 chlorinator on the prevalence of diarrheal disease in Nicaragua.^22,23^ CTI found that, among all communities with bacteria (Enterobacteriaceae family, tested via Hach Pathoscreen) present upstream of the passive chlorinator, no communities had bacteria present downstream of the chlorinator. On average, health centers in communities with CTI-8 passive chlorinators reported a decreased prevalence of diarrhea in comparison to communities without CTI-8 passive chlorinators (61% lower prevalence of disease).^23^ EOS compared rates of diarrheal disease in the years before and after installation of 70 CTI-8 chlorinators. They found that health centers reported a lower prevalence of diarrheal disease (49% reduction) after device installation.^22^ While the rapid global deployment of passive chlorinators is motivated by the goal to achieve universal access to safely managed drinking water, additional health impact studies in high-disease burden settings would be valuable to determine their cost effectiveness in reducing adverse health outcomes. The findings of these studies could motivate government policy makers and funders to invest in and support the deployment of passive chlorinators.^40^

### 2.3 Implementation Settings

The majority of passive chlorination studies reviewed here were conducted in rural communities, with only 15% (4/27) of the evaluations conducted in urban or peri-urban settings.^32,33,38,48^ We identified 27 passive chlorinator evaluations conducted across 16 countries, in communities ranging in size from 183 to 3000 people (Table 2). Several passive chlorinators were evaluated in settings outside of the traditional community settings. In Tanzania, Marcenac et al.^39^ installed Aquatabs Flo in 9 rural healthcare facilities in cholera hotspots. Aquatabs Flo provided water with a mean FCR between 0.3 and 0.9 mg/L,^39^ suggesting that passive chlorinators could provide safe drinking water in a relatively understudied but critical implementation setting. These initial results were reported under idealistic research conditions, during which systems could be repaired or adjusted as necessary. However, healthcare facilities typically have staff present who could be trained to manage the long-term maintenance and operational needs of passive chlorinators such as regular FCR monitoring.

Due to their compatibility with pressurized or gravity-fed water distribution systems, passive chlorinators may also be useful in humanitarian relief settings and across the transition from refugee camps to longer-term communities. Three studies observed passive chlorinators installed in post natural disaster, conflict, and humanitarian response settings in Haiti,^44^ Bangladesh,^45^ and South Sudan.^35^ However, the chlorinators in all three studies either provided insufficient FCR for disinfection^35,45^ or were entirely in disrepair within 2 years of installation,^44^ demonstrating the importance of having a committed organization to ensure the proper operation and maintenance of devices (i.e., regular chlorine monitoring and refills).^44^ In natural disaster, conflict, and complex emergency settings, where the goal is often to provide as many individuals with treated water as possible, long-term monitoring and management systems may be overlooked or difficult to achieve. Additional considerations may also be required to determine ideal dosing and operating requirements for emergency settings in comparison to community settings because of the inherent instability and potential for prolonged or repeated periods of natural disaster or conflict.^35,53^

## 3 Limitations and Priority Areas for Future Research

**Figure 1:**
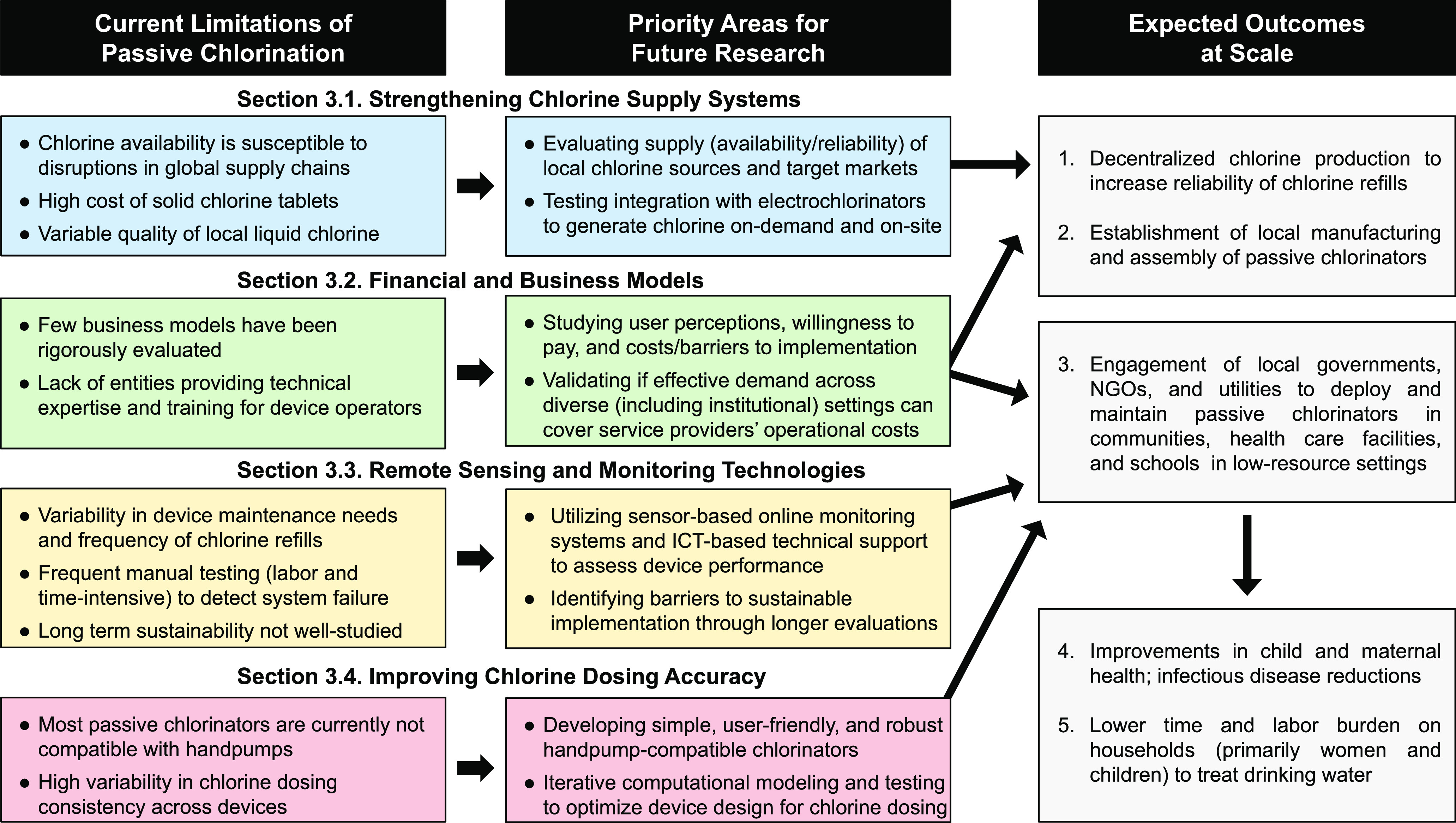
Pathways to address the current limitations of passive chlorination and achieve expected outcomes at scale. The scaling framework suggests methods to strengthen chlorine supply chains, develop and evaluate financial and business models, apply remote sensing and monitoring technologies, and improve chlorine dosing.

The sustained efficacy, effectiveness, and long-term adoption of passive chlorinators is dependent on having viable financial and business models, compatible infrastructure, consistent and accurate chlorine dosing, and reliable access to high-quality chlorine supplies. Here, we discuss the limitations of existing passive chlorinators (Figure 1), potential research directions to support the scaled deployment of passive chlorinators, expected outcomes at scale (Figure 1), and recommended standard indicators for reporting in future evaluations (Table 3).

**Table 3:**
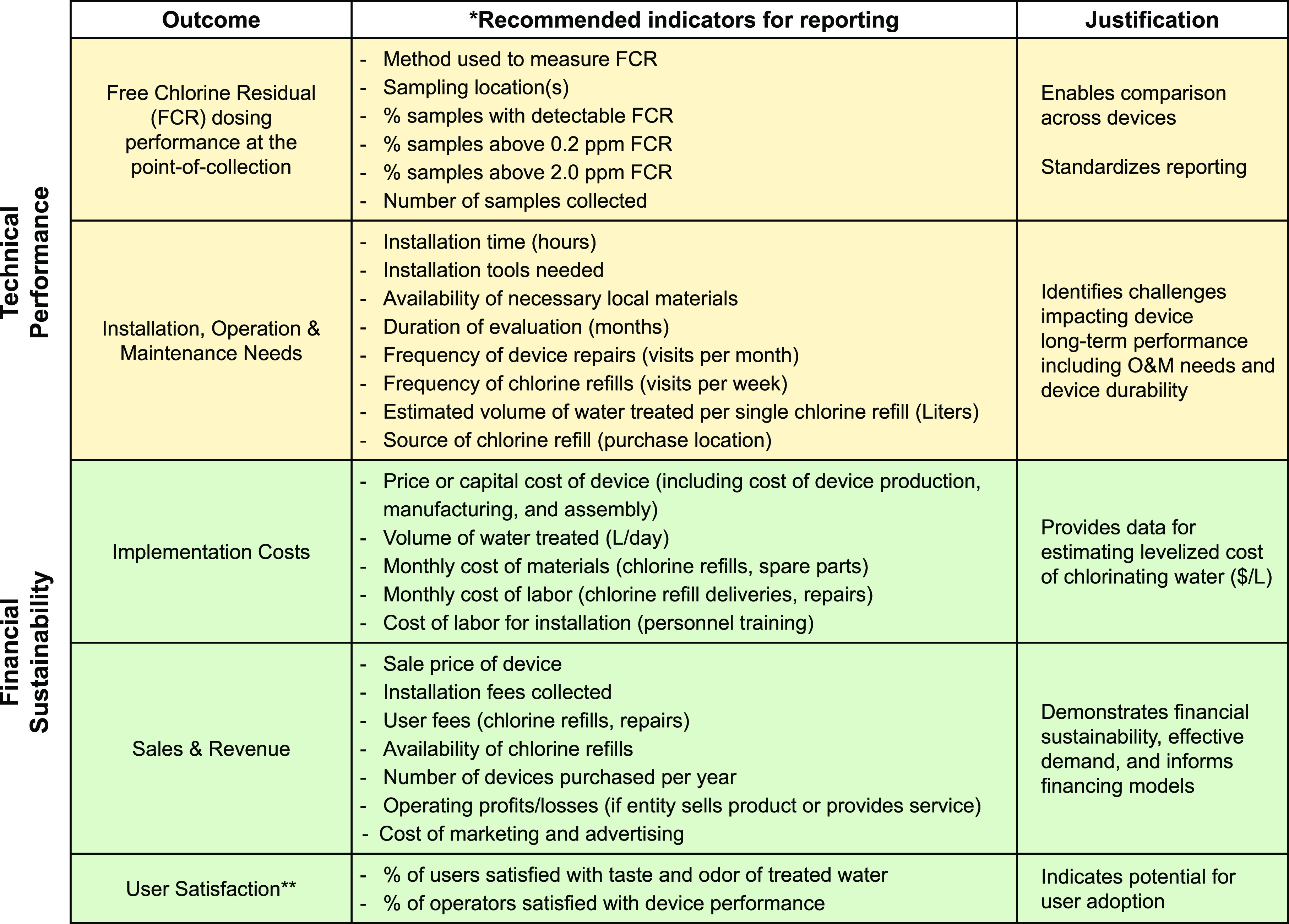
Recommended Standard Indicators for Reporting Future Evaluations of Passive Chlorinators^a^

a Legend: (*) all of the recommended indicators may not be relevant for every evaluation or study; (**) although we recommend measuring user satisfaction as an indicator, we recognize that user and operator surveys may not always be feasible and thus will be a lower priority in some cases.

### 3.1 Strengthening Chlorine Supply Systems

Adequate disinfection capacity and device operation depends on the procurement of high-quality chlorine and chlorine testing supplies. Devices requiring chlorine in the form of proprietary tablets or cartridges that need to be imported to low-income markets can dramatically increase the cost and difficulty of obtaining refills for passive chlorinators. One study of tablet chlorinators installed in Haiti noted that chlorine tablet procurement limited the long-term sustained use of passive chlorinators in communities.^44^ Dossegger et al.^21^ found that although some chlorine tablet sizes were locally available in Uganda, other sizes had to be imported. Some chlorinators also required refills using prepackaged or proprietary cartridges instead of generic tablets or liquid chlorine. The ability to use different chlorinators thus depends on the reliable availability of the “correct” type of chlorine. Liquid chlorinators may have an advantage, as some studies indicate that liquid chlorine is easier to find and purchase in resource-constrained settings and can be more easily produced locally.^21,48^

Solid chlorine is subject to changes in the global supply chain, as are testing supplies, which can significantly affect operating costs in the case of sudden geopolitical, climate, or global health disruptions. Even in high-income countries, sudden changes such as increased demand of chlorine due to the COVID-19 pandemic or manufacturing incidents (e.g., August 2020 chlorine production facility fire in Louisiana, USA)^54^ have caused temporary chlorine supply shortages, driving up the market price of chlorine.^55^ The global market remains sensitive to chlorine tablet shortages in high-income countries, indicating a need for increased local production capacity in low-income markets. We recommend that future evaluations on passive chlorinators report the cost and availability of chlorine used in the study (i.e., where the chlorine was procured to understand if it was locally available for purchase or imported) (Table 3). An additional research priority should be to identify regional or national chlorine supply flows, particularly in places where chlorinators are being or are likely to be implemented.

Although electrochlorinators^56−62^ were not evaluated in our review because of their external power requirement to generate chlorine from salt water, they are particularly important for their potential to increase local access to high-quality liquid chlorine in resource-constrained settings.^63^ While electrochlorinators substitute the problem of direct chlorine procurement with the requirement of electricity, continuous power is not necessary if sufficient chlorine can be generated on site when electricity is available or is generated by solar power. Although some studies have evaluated the pairing of electrochlorinators with community-level manual chlorination methods,^58,60^ continued research on the efficacy and economic feasibility of joint implementation with passive chlorinators would be valuable, particularly at health care facilities, where there are additional uses for chlorine (e.g., surface disinfection, hand cleaning). Making progress toward UN-SDG 7 (increasing access to affordable energy) could synergistically advance access to clean drinking water (UN-SDG 6.1) by making electrochlorinators more affordable and accessible.

### 3.2 Financial and Business Models

While very few studies have examined the financial viability of passive chlorination, there has been demonstrated effective demand for passive chlorinators among water kiosk owners and apartment landlords in some resource-constrained settings.^36,47^ Two evaluations specifically measured the effective demand for passive chlorinators coupled with an option for financing. Powers et al.^47^ evaluated the financial viability of leasing the Stanford-MSR Venturi chlorinator to 7 water kiosk owners in Kisumu, Kenya, in urban (1), peri-urban (2), and rural (4) areas. Researchers offered 4 service packages to kiosk owners, in order of increasing price: lease, lease + chlorine delivery, lease to own, and lease to own + chlorine delivery. At the end of the 6-month trial period, 6 out of 7 kiosk owners completed all of their service payments, and 5 of the 7 kiosk owners chose to purchase the chlorinator, including 2 who had previously chosen a leasing package. Given the option to purchase chlorinated or unchlorinated water (with water price varying by kiosk), kiosk users reported purchasing chlorinated water 66% of the time, suggesting that end users were willing to pay for treated water. This evaluation indicates that there is effective demand among kiosk owners in Kenya for passive chlorination, lending support to passive chlorination being economically viable for smaller-scale entrepreneurial and management structures.

Another effective demand study by Smith et al.^36^ in low- and middle-income communities in Dhaka, Bangladesh, found that, although some landlords are willing to pay for passive chlorinators (specifically Aquatabs Flo), further financial incentives may be required to ensure wider use and sustained payments for passive chlorination. The researchers offered landlords passive chlorinators to treat apartment building water supply systems and used Becker–DeGroot–Marschak (BDM) auctions (see below for additional details about this method) to elicit willingness to pay and monthly service payments. They evaluated multiple indicators of effective demand, including sustained payment for the chlorinator and maintenance services. Landlord effective demand for in-line chlorination was similar to or greater than that for POU treatment products and manual chlorine dispensers previously documented among Dhaka households. Ultimately, 33% of landlords in middle-income communities and 9% of landlords in low-income communities paid for the passive chlorinators for the full duration of the 1-year study period. Interestingly, most landlords did not attempt to pass on their own increased expenses for the passive chlorinator device by charging tenants higher utility costs or rent. In addition to understanding the motivations and behaviors of landlord or community-level water managers,^36^ additional research is needed to determine the factors driving user willingness to pay.^36^

Overall, these evaluations suggest that passive chlorination could potentially be a financially sustainable option for providing disinfected drinking water. Smith et al.^40^ and Powers et al.^48^ highlight the importance of financing models that account for the financial burden placed both on the end user and on the group managing the passive chlorinator (e.g., landlords or kiosk owners in these cases). In one study in Uganda, researchers estimated that, on the basis of the revenue for the types of gravity-driven membrane kiosks where passive chlorinators were implemented and evaluated, a cost of lower than $0.10/1000 L of drinking water chlorinated is reasonable and viable for continued profitability.^21^ Although passive chlorination appears to be relatively low cost, it is also clear that additional financial considerations beyond simply the low cost of chlorine are necessary for ensuring equitable access to and sustained operation of these technologies (Figure 1 and Table 3).

Evaluations of water treatment interventions in resource-constrained settings highlight the importance of sustained local stakeholder engagement and technical expertise for long-term and appropriate device operation, maintenance, and monitoring. In particular, community-scale water disinfection through passive chlorinators requires a designated person or group of people to regularly monitor FCR and refill solid or liquid chlorine.^28,48^ Additionally, passive chlorination at the community scale does not address the potential for recontamination; thus, adequate dosing at or prior to the POC still should be paired with safe water storage practices. More evaluations are necessary to determine whether and how local NGO and community-level management and support models can effectively address these challenges and achieve financial sustainability through subsidized service or user fee recovery models (Table 3).^28,44^ Strategies to sustainably finance this type of long-term service delivery are critical for scaling up passive chlorination.

A few studies, along with other evaluations on rural water services and sustainability, have suggested that community-level support from an NGO or internal management structure is critical for long-term effectiveness.^17,44,64^ Rayner et al.^44^ revisited passive chlorinators 2 years post-installation and found that chlorinators were likely to fail without community-level operation and management.^44^ Crider et al.^17^ monitored passive chlorination technologies over the course of a full year, allowing for an evaluation of sustained use across changing seasons. They found that, while monitoring was sustained, over 74% of POC samples across both types of chlorinators evaluated had greater than 0.1 mg/L FCR.^17^ In general, evaluations spanning longer time periods are needed to better understand the long-term sustainability and effectiveness of passive chlorinators. In most studies discussed in this review, chlorinators were installed only for the duration of the study and then removed.^21,38,39^ For studies evaluating existing chlorinators installed prior to the evaluation, the study period was often not more than a few weeks to a few months.^20,28,29^ The results from our NGO survey suggest that longer-term evaluations could be conducted for many chlorinators that have already been deployed and are currently in use in resource-constrained communities.

Furthermore, there is a clear need to develop site-specific business models (or subsidized service delivery models) for both institutional and community settings^60^ and to evaluate community capacity to support passive chlorination.^40^ Economic feasibility studies conducted on other forms of community water treatment, such as solar-powered electrochlorination, provide replicable methods for evaluating the scalability of profits across international markets.^60^ While an effective demand for passive chlorination has been demonstrated at community water points (e.g., among kiosks and landlords), there is a need for research on financially sustainable distribution models for institutional settings (e.g., schools, healthcare facilities). A recent study by Marcenac et al.^39^ produced preliminary results (in a controlled research setting) suggesting that passive chlorinators can work effectively in rural Tanzanian healthcare facilities. Another study by Ribeiro et al.^62^ (not included in this review because the chlorinator was not passive) concluded that having designated staff members to maintain and operate a manual chlorinator effectively improved its performance in a school setting.^62^ Implementation research and collaborative monitoring-based research approaches^65^ offer a pathway to characterize the technical efficacy and financial viability of passive chlorinators, without requiring significant additional resources to launch large-scale research studies from scratch (Table 3).

Currently, it is difficult to estimate a levelized cost (in $/L) of water treated by passive chlorinators. Information regarding the operation and maintenance cost (i.e., chlorine replacement, device maintenance, personnel, etc.) was unavailable for a majority of the devices in Table 1. Only four studies included in this review explicitly discussed financial viability, and only Powers et al.^48^ and Smith et al.^40^ evaluated implementation models for sustained financing and willingness and ability to pay for services. Two well-established methodologies in economics to evaluate individual or institutional ability and willingness to pay include take-it-or-leave-it (TIOLI) offers or BDM auctions. In TIOLI experiments, different users are offered the intervention (e.g., passive chlorinators with or without chlorine refills) at randomized prices (potentially including “free”) and a demand curve is drawn on the basis of the fraction of users in the field who are willing to pay each price. BDM auctions ask the user to bid the price they would be willing to pay for the product; if the bid is above a randomly assigned price, the respondent purchases the product at the assigned price, while if the bid is below the price, they pay nothing and receive nothing. This method elicits true willingness to pay because there is no incentive to lie about the price one is willing to pay.^40^ However, it is worth noting that both of these methodologies require products to be actually sold to ensure customers are both willing and able to pay a certain price.

Another helpful framework for characterizing financial viability is presented in a recent paper published by Amrose et al.,^68^ which summarizes the costs of different approaches to remediate chemical contaminants in drinking water in low-resource settings. We recommend that future groups evaluating passive chlorinators report information on implementation costs, sales and revenue (where applicable), and user satisfaction surveys (Table 3). Examples of specific costs that should be collected include staff transport costs to deliver chlorine refills, material cost of chlorine refills, labor costs of personnel training and time, and marketing and advertising costs to increase device adoption. Ideally, because chlorine consumption in passive chlorinators is directly proportional to the flow rate, recurring material costs can be estimated by implementing organizations and communities. However, when chlorine costs are reported, fluctuating local and global markets and variable price points of proprietary chlorine refill cartridges sold by different local distributors must also be considered.

### 3.3 Remote Sensing and Monitoring Technologies

Although automated sensors are regularly used to monitor water quality in wastewater and drinking water utilities in high-income countries, their application to track drinking water quality in LMICs is not as common.^67^ Increased affordability of mobile phone technologies has supported the deployment of remote monitoring systems to assess the functionality, use, and effectiveness of WASH interventions.^67,68^ Examples of remote sensing device applications in the WASH sector include continuous monitoring of handpump functionality,^69^ usage patterns of latrines,^70^ and usage of hand-washing stations.^71^ Machine-learning platforms have also been demonstrated to improve handpump functionality by using daily monitoring data to predict and respond to upcoming maintenance needs.^69^ Similarly, outside of resource-constrained settings, machine learning and advanced data analytics have been used at municipal water utilities^72^ to detect anomalies,^73^ classify known events and irregularities, and predict future trends in water quality. These monitoring systems could be integrated into local, regional, and national agencies responsible for establishing standards and legislation and ensuring that systems routinely deliver safe water.^49^ Additionally, sensor-based monitoring and evaluation systems can be used to collect and analyze data to improve long-term service delivery (Figure 1).

We recommend that researchers investigate the pairing of water quality sensors and data processing tools to improve the reliability of measuring chlorine levels, to detect dosing irregularities and trigger alerts for maintenance needs of passive chlorinators, and to reduce monitoring costs. Current real-time chlorine sensors are expensive and not yet well adapted to long-term *in situ* monitoring.^67^ Improved sensor development and use of sensors to measure alternative water quality parameters such as oxidation reduction potential (ORP) and pH^74^ as proxies for estimating chlorine could lower the cost of real-time monitoring. Additionally, information and communication technologies (ICT) offer implementing organizations and technicians the potential to use SMS messaging for training, monitoring, and sending maintenance reminders. Real-time monitoring and early detection of system failures could increase the consistency of adequate FCR dosing, which currently depends on community members and technicians. In the case of most tablet chlorinators^28^ and liquid dosers, the device operator would also be responsible for adjusting dosing configurations. Additionally, testing for FCR or the presence of*E. coli* requires additional expertise and materials, which can further burden users and increase cost.^75^ Addressing these issues requires acknowledging the inherent difficulty of integration between local water utility governance structures and decentralized chlorine monitoring. Though many countries have regulations for drinking water chlorination, the governing bodies overseeing those regulations may not be the entities maintaining and operating the passive chlorinators. Pairing water quality monitoring sensors with ICT-based support could bolster the capacity of water service providers and passive chlorinator operators.

The WHO chlorination guidelines recommend FCR greater than or equal to 0.5 mg/L throughout the distribution system and at least 0.2 FCR at the POC or point of delivery for a piped infrastructure. For household POU water treatment, the FCR should be greater than 0.2 mg/L but not exceed 2.0 mg/L.^49^ The studies included in this review measured FCR at the tap, directly after chlorination, in stored water, or throughout a distribution network. A recent study by Ali et al.^55^ on household water safety in humanitarian and emergency WASH settings took paired chlorine measurements at the point of disinfection (i.e., at the passive chlorinator) and along the distribution network. Using these paired measurements, they built a model predicting an ideal initial chlorine dose to maintain an adequate FCR in the water at the points of collection and use.^55^ To further validate particular dosing strategies for different passive chlorinators used by communities, additional studies could pair FCR measurements taken at the point of disinfection/collection with measurements taken from stored water or points downstream of disinfection. This approach could help inform site-specific strategies to optimize initial chlorine dosing to maintain proper FCR until the POU across different types of implementation settings. In some instances, the infrastructure guidelines of at least 0.2 mg/L at the point of delivery may be sufficient, while in other settings a greater residual may be necessary. Therefore, there is a need to develop a standardized approach for applying existing guidelines and comparing effectiveness metrics for passive chlorinators across settings. Specifically, we recommend that future evaluations consider both free chlorine residual as well as metrics related to installation, operation, and maintenance (Table 3). Free chlorine should be measured at the POC, and in some cases, it might be valuable to also measure FCR in stored water at the POU. Researchers should report the method of measurement used, the proportion of samples with detectable FCR, and the proportion of samples with FCR between 0.2 and 2 ppm (Table 3).

### 3.4 Improving Chlorine Dosing Accuracy

We note that there is limited evidence that existing passive chlorinators are compatible with (i.e., can dose effectively) manual handpumps. Three passive chlorinators reviewed here (PurAll 50H, Nirapad Pani, and Zimba) were all explicitly designed for automated dosing at the outflow of handpumps. PurAll 50H^45^ did not provide consistent chlorine dosing (only 44% of samples were >0.2 mg/L FCR), and Nirapad Pani^32^ remains uncommercialized. While Zimba^33^ showed promising results (100% of samples collected were between 0.2 and 2.0 mg/L FCR), it is a batch chlorination device that requires 10 L of water to be pumped per batch. Although handpumps provide critical access points by extracting water from groundwater aquifers, they are prone to microbial contamination and often do not meet the criteria for safely managed drinking water.^76,77^ Given that, as of 2010, 1.3 billion people obtained drinking water from handpumps^78^ in both urban and rural settings, the development or adaptation of passive chlorinators that can dose accurately and consistently at handpump outflows would substantially increase the target market for passive chlorination.

Variability in flow rates, source water chlorine demand, and solid chlorine tablet dissolution rates can change the level of effectiveness of passive chlorinators across implementation settings. While some passive chlorinators automatically adjust the chlorine dose on the basis of influent water flow rate (e.g., Stanford-MSR Venturi, Nirapad Pani, AguaClara), others rely on manual dose adjustment using a bypass valve or other mechanism (Table 1). As a result of these differences in dosing mechanisms, flow rates,^21,39,42^ and the systems in which passive chlorinators are installed, dosing consistencies vary across devices (Table 2). Furthermore, installing passive chlorinators upstream of the POC with intermittent water supplies^7^ can complicate dosing because stagnant water can rapidly dissolve chlorine. Evidence for compatibility with an intermittent supply exists for a subset of the passive chlorinators reviewed here.^17,33,38,48^ Other passive chlorinators presented in Table 1 require further development or evaluation of system requirements to maintain ideal dosing, particularly in intermittent and pressurized systems.

Prior to and in parallel with direct field testing, computational fluid dynamic (CFD) modeling and laboratory-based evaluation can be used to optimize passive chlorinator dosing consistency and to modify existing chlorinators for increased compatibility with new supply systems. Historically, CFD tools have been used to model complex processes in a variety of fluid systems across disciplines and industrial processes, which are otherwise challenging to investigate experimentally.^79,80^ Martin^42^ used CFD models in conjunction with laboratory-scale models of Fluidtrol Processes Chlorinator, an erosion chlorinator currently deployed in Cange, Haiti. The authors investigated the effect of inlet and outlet position on the outlet FCR concentration and used their findings to enhance the system to use the most effective positioning in real time.^42^ A study on the Aquatabs Flo^37^ used dye tracer assessments to determine that the dose changed significantly when the passive chlorinator was improperly positioned. We recommend that future research utilize CFD modeling and iterative laboratory-scale testing prior to and between field pilots to choose design parameters that will improve the dosing consistency of passive chlorinators adapted for novel implementation settings and systems.

## 4 Limitations of Chlorine as a Disinfectant

For any water treatment technology reliant on chlorine, it is critical to consider the potential formation of DBPs during the disinfection process.^81^ Maintaining precise chlorine dosing has the added benefit of potentially mitigating the formation of DBPs. There are several classes of DBPs associated with chlorination, including haloacetic acids (HAAs) and trihalomethanes (THMs). THMs have been linked to a potential increased risk of bladder cancer as well as other negative reproductive health outcomes,^82,83^ although the health risk posed by exposure to waterborne pathogens is widely thought^84−86^ to outweigh the risk posed by exposure to DBPs. Further, research studies have shown that THM formation in LMIC waters is below the WHO standards for THMs and other currently regulated DBPs.^84,85^ However, there is emerging evidence on other DBPs that may indicate a need for further testing at chlorine dosage levels used in actual passive chlorinator programs. For example, Furst et al. conducted a study^87^ in India and noted that THMs, the class of DBPs most often used to estimate DBP prevalence, were poorly correlated with more toxic classes of DBPs, which often are not directly measured but can have more adverse health impacts. This finding, in addition to the lack of current research on THMs and passive chlorinators, further emphasizes the importance of studying a variety of DBPs in resource-constrained settings.

The natural taste and odor of higher chlorine doses can make users adverse to drinking chlorinated water. Studies on the taste of chlorinated water have indicated that users will refuse to drink chlorinated water above a certain concentration threshold due to the adverse taste.^88,89^ Free chlorine dose taste thresholds vary across settings and in many cases^89,90^ are lower than the upper limit of the WHO target chlorine dose to achieve adequate disinfection in household POU scenarios (0.2–2.0 mg/L). This suggests the need for site-specific taste threshold research, particularly in settings in which taste thresholds are not available. However, the dosing precision and accuracy reported for many passive chlorinators included in this review suggest that it would be possible to maintain chlorine doses within both WHO disinfection guidelines and taste aversion thresholds, optimizing both user acceptability and the health benefits of drinking water chlorination. For example, only 14% of respondents in the treatment group of a blinded passive chlorination trial in Dhaka, Bangladesh, thought they knew whether or not they were receiving chlorinated drinking water.^89^ Despite the low chlorine dosing in this trial (∼0.4 mg/L), there was still a documented health benefit for children (i.e., reduced diarrhea prevalence).

No studies examining DBP formation and end user acceptance of water treated by passive chlorination currently exist. Taste aversion can significantly influence not only users’ willingness to consume the drinking water but also their potential demand for chlorinated water and, in turn, willingness to pay.^89^ A recent study by Smith et al.^91^ in urban Bangladesh indicates that maintaining a chlorine dose within the range of taste/odor and disinfection thresholds can significantly reduce the risk of waterborne disease while also minimizing disinfection-byproduct consumption because users are more likely to consume the chlorinated water and less likely to turn to alternative water sources. In order to design and test future financial implementation models, it will be important to ensure that the water being produced by passive chlorinators falls within reasonable taste thresholds and is free of chemicals that could have long-term chronic health effects (e.g., arsenic and fluoride). We recommend future studies characterize and quantify the DBPs produced by passive chlorinators and examine the effect of DBPs and taste aversion on user perceptions and acceptability, particularly in settings with poor water quality and high organic loads.

Pathogens such as*Cryptosporidium* and*Giardia* found in natural water sources have a high disease burden in low-income settings^92^ and are difficult to inactivate with standard chlorine doses appropriate for human consumption and contact times (i.e., average water storage times). Orner et al.^28^ found that tablet chlorinators without storage tanks did not meet the necessary dose or contact time requirements for inactivation of *Giardia*. Similarly, Brignoni^26^ observed that tablet chlorinators were unable to deliver a chlorine dose high enough to eliminate *Giardia* in community settings in Puerto Rico. When possible, the installation of a storage tank after a passive chlorinator can increase contact time and the potential to inactivate these pathogens. However, regardless of the initial dose or the addition of a storage tank, passive chlorinators may be unable to provide sufficient disinfection for protozoan pathogens, which can be removed by filtration or inactivated by boiling, ozonation, or UV irradiation.

## 5 Limitations of This Review

We note that there are several limitations to this review. First, we only included passive chlorinators implemented in LMICs. Technologies that have only been evaluated in high-income countries with applications in low resource settings may have been missed. Second, we did not systematically assess the quality of the papers included in this review. Finally, there was a lack of published evidence on the costs and financial viability of existing passive chlorination devices. This motivated the recommended indicators for reporting we have given in Table 3, to encourage researchers and program implementers to help fill this gap by standardizing future evaluations and comparisons of passive chlorinators.

## 6 Conclusion

A large variety of passive chlorinators are now available that do not require electricity, automatically dose chlorine in different forms, are compatible with infrastructure in resource-constrained settings, and are capable of providing drinking water that meets WHO guidelines for FCR and*E. coli*contamination. We reviewed 27 different passive chlorinators evaluated in 27 peer-reviewed studies, theses, NGO reports, and field pilots conducted across 16 countries in numerous communities and settings at multiple scales including households, community shared water collection points, and community-wide piped distribution networks.

In comparison to manual chlorination methods, automatic dosing by passive chlorinators can reduce the burden placed on end users to treat water and continually monitor or iteratively manage FCR, leading to increased adherence and improved water quality and consequent health benefits.^28,48,60,66^ Because passive chlorinators can be installed in existing water delivery systems, they allow users of these systems to maintain their regular water collection and management practices.^32,33,38^ Passive chlorinators can be installed at different points along a water distribution system where they are needed, allowing them to be compatible with taps and distribution networks and to take advantage of the contact time provided by storage tanks. Passive chlorinators can also be adapted to a variety of flow rates and flow regimes such as those found in gravity-fed and pressurized systems. Most passive chlorinators are also compatible with intermittent water supplies,^17,32,33,48^ which currently serve approximately 1 billion people worldwide.^93^

Many passive chlorinators can dose precisely and accurately over a range of FCR concentrations ideal for many settings, although there is variability in dosing consistency across studies and technologies and not all technologies were assessed with equivalent methods. Chlorine dosing precision is an important factor in technology selection, because an effective chlorinator must dose reliably within a range that maintains a taste and odor that is acceptable to end users. Multiple studies have indicated that users across different regions have varying acceptability thresholds for the taste and odor of chlorinated water, and that may affect site-specific dosing strategies.^89,90^ For example, one study conducted in Bangladesh^38^ found that Aquatabs Flo could reliably dose FCR between 0.1 and 1.2 mg/L for 85% of samples collected, staying below the maximum acceptable dose of approximately 1.2 mg/L identified in the same setting.^89^

Our analysis of the economic and financial evaluation studies demonstrated that passive chlorinators typically have an average capital cost of $140 (and as low as ∼$3). Some lower-cost tablet chlorinators^27,28,46^ and liquid dosers^21^ reviewed in this paper can be constructed using affordable and locally available materials such as PVC pipe. The passive chlorinators reviewed here require no electricity to operate, and when chlorine is available and locally accessible, operational costs have the potential to be low, as chlorine is one of the lowest-cost disinfectants.^94^

Our key recommendations for future research to inform if the widespread deployment and adoption of passive chlorinators are warranted are as follows: (i) evaluate local chlorine availability and strengthen supply chains through decentralized chlorine production and integration with electrochlorination, (ii) develop and test new financial and business models with consideration of end-user perceptions (e.g., taste/odor and DBPs in chlorinated water), willingness to pay, and effective demand, (iii) apply remote monitoring and sensing technologies integrated with data processing tools to increase chlorine dosing accuracy, and (iv) develop passive chlorinators compatible with handpumps. The 2021 JMP database suggests that an estimated 3.9 billion people in LMICs globally are currently using piped improved drinking water supplies, which are compatible with passive chlorinators.^95^ In systems that already address chemical contamination and provide continuous on-premises water access, passive chlorinators represent a promising strategy toward achieving SDG 6.1 by elevating drinking water into the safely managed status.

## Supporting Information Available

Additional details related to the literature search strategy using PubMed, Scopus, and Proquest databases and the survey form distributed to NGOs (PDF)
