# Supplementary material for: Passive In-Line Chlorination for Drinking Water Disinfection: A Critical Review
Source: Environ Sci Technol. 2022 Jun 14;56(13):9164–81. doi: 10.1021/acs.est.1c08580 (PMC9261193; doi:10.1021/acs.est.1c08580)
Supplement: Supplementary file 1 — es1c08580_si_001.pdf [file es1c08580_si_001.pdf]

Supporting Information

Passive in-line chlorination for drinking water disinfection in resource-constrained settings: A Critical Review

Megan Lindmark[τ], Katya Cherukumilli[τ], Yoshika S. Crider, Perrine Marcenac, Matthew Lozier, Lee Voth-Gaeddert, Daniele S. Lantagne, James R. Mihelcic, Qianjin Zhang, Craig Just[α] *, Amy J. Pickering[α] *

τ These authors are co-first authors
α These authors contributed equally to the manuscript

*Correspondence to: Amy J. Pickering, pickering@berkeley.edu; Craig Just, craig-just@uiowa.edu

Table of Contents

1. Search Strategy Method..
    PubMed Search which excluded developed countries S2
    Revised PubMed search which included developing countries S3
    Scopus search which excluded developed countries S5
    Revised Scopus search which included developing countries S5
    ProQuest Dissertations & Theses Global search S6
    Flow Diagram........ S7
2. NGO Survey S8

1. Search Strategy Method

Search strategies were developed with the assistance of an engineering and informatics librarian with expertise in searching for systematic reviews. Original search strategies, including both index and keyword methods to maximize sensitivity, were conducted on March 19, 2021 in the following databases: PubMed, Scopus, and ProQuest Dissertations and Theses Global. For search terms, the first and second main concepts were combined with the AND operator: one to designate the topic of chlorinator and the other to designate the topic of water treatment; the third concept of wastewater were excluded in the search using the NOT operator. Geographic regions of developed countries (North American, European countries, East Asian and Oceania countries) were excluded in the search using NOT operators. The English, Chinese, French and Spanish filters were applied. Total yield and duplicate count can be found in the flow diagram. Duplicates were managed primarily utilizing reference management software. Patent search strategies were also conducted on March 23, 2021 in the following databases: Patentscope, USPTO and Lens. Updated search strategies, in which geographic regions of developing countries were included using the AND operator, were conducted on May 15, 2021, identifying additional papers. Original search strategies were conducted again on the same day.

PubMed Search which excluded developed countries

S1 = “chlorinator” [Text Word] OR “chlorinate” [Text Word] OR “chlorination” [Text Word] OR “chlorine doses” [Text Word] OR “chlorine dosing” [Text Word]

S2 = "Water Quality "[MeSH] OR “drinking water” [MeSH] OR “water quality” [Text Word] OR “drinking water” [Text Word] OR inline [Text Word] OR in-line [Text Word] OR “water treatment” [Text Word]

S3 = S1 AND S2

S4 = “waste water” [MeSH] OR sewage [MeSH] OR wastewater [Text Word] OR “waste water” [Text Word] OR sewage [Text Word]

S5 = S3 NOT S4

S6 = Europe [MeSH] OR Canada [MeSH] OR United States [MeSH] OR Greenland [MeSH] OR Japan [MeSH] OR “Republic of Korea” [MeSH] OR Australia [MeSH] OR “New Zealand” [MeSH] OR USA [Title/Abstract] OR “United States” [Title/Abstract] OR Canada [Title/Abstract] OR Austria [Title/Abstract] OR Belgium [Title/Abstract] OR France [Title/Abstract] OR Germany [Title/Abstract] OR Liechtenstein [Title/Abstract] OR Luxembourg [Title/Abstract] OR Monaco [Title/Abstract] OR Netherlands [Title/Abstract] OR Switzerland [Title/Abstract] OR “United Kingdom” [Title/Abstract] OR UK [Title/Abstract] OR Spain [Title/Abstract] OR Portugal [Title/Abstract] OR Andorra [Title/Abstract] OR Greece [Title/Abstract] OR Italy [Title/Abstract] OR “Vatican City” [Title/Abstract] OR Japan [Title/Abstract] OR “Republic of Korea” [Title/Abstract] OR “South Korea” [Title/Abstract] OR Australia [Title/Abstract] OR “New Zealand” [Title/Abstract]

S5 NOT S6

Language limit to English, Chinese, French, Spanish

Revised PubMed search which included developing countries

Step 1 through 5 were the same as above. But Step 6 was as below:

S6 = Afghanistan [MeSH] OR Benin [MeSH] OR “Burkina Faso” [MeSH] OR Burundi [MeSH] OR “Central African Republic” [MeSH] OR Chad [MeSH] OR Congo [MeSH] OR Zaire [MeSH] OR Eritrea [MeSH] OR Ethiopia [MeSH] OR Gambia [MeSH] OR Guinea [MeSH] OR Guinea-Bissau [MeSH] OR Haiti [MeSH] OR “Democratic People's Republic of Korea” [MeSH] OR Liberia [MeSH] OR Madagascar [MeSH] OR Malawi [MeSH] OR Mali [MeSH] OR Mozambique [MeSH] OR Nepal [MeSH] OR Niger [MeSH] OR Rwanda [MeSH] OR “Sierra Leone” [MeSH] OR Somalia [MeSH] OR “South Sudan” [MeSH] OR Sudan [MeSH] OR Syria [MeSH] OR Tajikistan [MeSH] OR Tanzania [MeSH] OR Togo [MeSH] OR Uganda [MeSH] OR Yemen [MeSH] OR Angola [MeSH] OR Bangladesh [MeSH] OR Bhutan [MeSH] OR Bolivia [MeSH] OR “Cabo Verde” [MeSH] OR Cambodia [MeSH] OR Cameroon [MeSH] OR Comoros [MeSH] OR Congo [MeSH] OR “Cote d'Ivoire” [MeSH] OR Djibouti [MeSH] OR Egypt [MeSH] OR “El Salvador” [MeSH] OR Swaziland [MeSH] OR Eswatini [MeSH] OR Ghana [MeSH] OR Honduras [MeSH] OR India [MeSH] OR Indonesia [MeSH] OR Kenya [MeSH] OR Kiribati [MeSH] OR Kyrgyzstan [MeSH] OR Laos [MeSH] OR Lesotho [MeSH] OR Mauritania [MeSH] OR Micronesia [MeSH] OR Moldova [MeSH] OR Mongolia [MeSH] OR Morocco [MeSH] OR Myanmar [MeSH] OR Burma [MeSH] OR Nicaragua [MeSH] OR Nigeria [MeSH] OR Pakistan [MeSH] OR “Papua New Guinea” [MeSH] OR Philippines [MeSH] OR “Sao Tome and Principe” [MeSH] OR Senegal [MeSH] OR Melanesia [MeSH] OR Sudan [MeSH] OR Timor-Leste [MeSH] OR Tunisia [MeSH] OR Ukraine [MeSH] OR Uzbekistan [MeSH] OR Vanuatu [MeSH] OR Vietnam [MeSH] OR “Middle East” [MeSH] OR Zambia [MeSH] OR Zimbabwe [MeSH] OR Albania [MeSH] OR Algeria [MeSH] OR “American Samoa” [MeSH] OR Argentina [MeSH] OR Armenia [MeSH] OR Azerbaijan [MeSH] OR Belarus [MeSH] OR Belize [MeSH] OR “Bosnia and Herzegovina” [MeSH] OR Yugoslavia [MeSH] OR Botswana [MeSH] OR Brazil [MeSH] OR Bulgaria [MeSH] OR China [MeSH] OR Colombia [MeSH] OR “Costa Rica” [MeSH] OR Cuba [MeSH] OR Dominica [MeSH] OR “Dominican Republic” [MeSH] OR Ecuador [MeSH] OR “Equatorial Guinea” [MeSH] OR Fiji [MeSH] OR Gabon [MeSH] OR Georgia [MeSH] OR Grenada [MeSH] OR Guatemala [MeSH] OR Guyana [MeSH] OR Iran [MeSH] OR Iraq [MeSH] OR Jamaica [MeSH] OR Jordan [MeSH] OR Kazakhstan [MeSH] OR Kosovo [MeSH] OR Lebanon [MeSH] OR Libya [MeSH] OR Malaysia [MeSH] OR Maldives [MeSH] OR Micronesia [MeSH] OR Mexico [MeSH] OR Montenegro [MeSH] OR Namibia [MeSH] OR “Republic of North Macedonia” [MeSH] OR Paraguay [MeSH] OR Peru [MeSH] OR Russia [MeSH] OR Samoa [MeSH] OR Serbia [MeSH] OR “South Africa” [MeSH] OR “Sri Lanka” [MeSH] OR “Saint Lucia” [MeSH] OR “Saint Vincent and the Grenadines” [MeSH] OR Suriname [MeSH] OR Thailand [MeSH] OR Tonga [MeSH] OR Turkey [MeSH] OR Turkmenistan [MeSH] OR Tuvalu [MeSH] OR Venezuela [MeSH]

OR Afghanistan [Title/Abstract] OR Benin [Title/Abstract] OR “Burkina Faso” [Title/Abstract] OR Burundi [Title/Abstract] OR “Central African Republic” [Title/Abstract] OR Chad [Title/Abstract] OR Congo [Title/Abstract] OR Zaire [Title/Abstract] OR Eritrea [Title/Abstract] OR Ethiopia [Title/Abstract] OR Gambia [Title/Abstract] OR Guinea [Title/Abstract] OR Guinea-Bissau [Title/Abstract] OR Haiti [Title/Abstract] OR “North Korea” [Title/Abstract] OR Liberia [Title/Abstract] OR Madagascar [Title/Abstract] OR Malawi [Title/Abstract] OR Mali [Title/Abstract] OR Mozambique [Title/Abstract] OR Nepal [Title/Abstract] OR Niger [Title/Abstract] OR Rwanda [Title/Abstract] OR “Sierra Leone” [Title/Abstract] OR Somalia [Title/Abstract] OR “South Sudan”

[Title/Abstract] OR Sudan [Title/Abstract] OR Syria [Title/Abstract] OR Tajikistan [Title/Abstract] OR Tanzania [Title/Abstract] OR Togo [Title/Abstract] OR Uganda [Title/Abstract] OR Yemen [Title/Abstract] OR Angola [Title/Abstract] OR Bangladesh [Title/Abstract] OR Bhutan [Title/Abstract] OR Bolivia [Title/Abstract] OR “Cabo Verde” [Title/Abstract] OR “Cape Verde” [Title/Abstract] OR Cambodia [Title/Abstract] OR Cameroon [Title/Abstract] OR Comoros [Title/Abstract] OR Congo [Title/Abstract] OR “Cote d'Ivoire” [Title/Abstract] OR Djibouti [Title/Abstract] OR Egypt [Title/Abstract] OR “El Salvador” [Title/Abstract] OR Swaziland [Title/Abstract] OR Eswatini [Title/Abstract] OR Ghana [Title/Abstract] OR Honduras [Title/Abstract] OR India [Title/Abstract] OR Indonesia [Title/Abstract] OR Kenya [Title/Abstract] OR Kiribati [Title/Abstract] OR Kyrgyzstan [Title/Abstract] OR “Kyrgyz Republic” [Title/Abstract] OR Laos [Title/Abstract] OR Lesotho [Title/Abstract] OR Mauritania [Title/Abstract] OR Micronesia [Title/Abstract] OR Moldova [Title/Abstract] OR Mongolia [Title/Abstract] OR Morocco [Title/Abstract] OR Myanmar [Title/Abstract] OR Burma [Title/Abstract] OR Nicaragua [Title/Abstract] OR Nigeria [Title/Abstract] OR Pakistan [Title/Abstract] OR “Papua New Guinea” [Title/Abstract] OR Philippines [Title/Abstract] OR “Sao Tome and Principe” [Title/Abstract] OR Senegal [Title/Abstract] OR Melanesia [Title/Abstract] OR “Solomon Islands” [Title/Abstract] OR Timor-Leste [Title/Abstract] OR Tunisia [Title/Abstract] OR Ukraine [Title/Abstract] OR Uzbekistan [Title/Abstract] OR Vanuatu [Title/Abstract] OR Vietnam [Title/Abstract] OR “Middle East” [Title/Abstract] OR “Gaza Strip” [Title/Abstract] OR Zambia [Title/Abstract] OR Zimbabwe [Title/Abstract] OR Albania [Title/Abstract] OR Algeria [Title/Abstract] OR “American Samoa” [Title/Abstract] OR Argentina [Title/Abstract] OR Armenia [Title/Abstract] OR Azerbaijan [Title/Abstract] OR Belarus [Title/Abstract] OR Belize [Title/Abstract] OR “Bosnia and Herzegovina” [Title/Abstract] OR Yugoslavia [Title/Abstract] OR Botswana [Title/Abstract] OR Brazil [Title/Abstract] OR Bulgaria [Title/Abstract] OR China [Title/Abstract] OR Colombia [Title/Abstract] OR “Costa Rica” [Title/Abstract] OR Cuba [Title/Abstract] OR Dominica [Title/Abstract] OR “Dominican Republic” [Title/Abstract] OR Ecuador [Title/Abstract] OR “Equatorial Guinea” [Title/Abstract] OR Fiji [Title/Abstract] OR Gabon [Title/Abstract] OR Georgia [Title/Abstract] OR Grenada [Title/Abstract] OR Guatemala [Title/Abstract] OR Guyana [Title/Abstract] OR Iran [Title/Abstract] OR Iraq [Title/Abstract] OR Jamaica [Title/Abstract] OR Jordan [Title/Abstract] OR Kazakhstan [Title/Abstract] OR Kosovo [Title/Abstract] OR Lebanon [Title/Abstract] OR Libya [Title/Abstract] OR Malaysia [Title/Abstract] OR Maldives [Title/Abstract] OR Micronesia [Title/Abstract] OR “Marshall Islands” [Title/Abstract] OR Mexico [Title/Abstract] OR Montenegro [Title/Abstract] OR Namibia [Title/Abstract] OR “Republic of North Macedonia” [Title/Abstract] OR Paraguay [Title/Abstract] OR Peru [Title/Abstract] OR Russia [Title/Abstract] OR Samoa [Title/Abstract] OR Serbia [Title/Abstract] OR “South Africa” [Title/Abstract] OR “Sri Lanka” [Title/Abstract] OR “Saint Lucia” [Title/Abstract] OR “St. Lucia” [Title/Abstract] OR “St. Vincent and the Grenadines” [Title/Abstract] OR “Saint Vincent and the Grenadines” [Title/Abstract] OR Suriname [Title/Abstract] OR Thailand [Title/Abstract] OR Tonga [Title/Abstract] OR Turkey [Title/Abstract] OR Turkmenistan [Title/Abstract] OR Tuvalu [Title/Abstract] OR Venezuela [Title/Abstract]

S5 AND S6

Filters applied were also the same as the original search strategies:

Language limit to English, Chinese, French, Spanish

Scopus search which excluded developed countries

S1 = TITLE-ABS-KEY (chlorinator OR chlorinate OR chlorination OR {chlorine doses} OR {chlorine dosing})

S2 = TITLE-ABS-KEY ({water quality} OR {drinking water} OR inline OR in-line OR {water treatment})

S3 = S1 AND S2

S4 = TITLE-ABS-KEY (wastewater OR {waste water} OR sewage)

S5 = S3 AND NOT S4

S6 = TITLE-ABS-KEY (USA OR {United States} OR Canada OR Austria OR Belgium OR France OR Germany OR Liechtenstein OR Luxembourg OR Monaco OR Netherlands OR Switzerland OR {United Kingdom} OR UK OR Spain OR Portugal OR Andorra OR Greece OR Italy OR {Vatican City} OR Japan OR {Republic of Korea} OR {South Korea} OR Australia OR {New Zealand})

S5 AND NOT S6

Subject Area Limit to Environmental Science

Keywords limit to "Chlorination", "Water Treatment", "Drinking Water", "Disinfection", "Chlorine", "Water Quality" (see the screenshots above)

Language Limit to English, Chinese, French and Spanish

Revised Scopus search which included developing countries

Step 1 through 5 were the same as above. But Step 6 was as below:

S6 = TITLE-ABS-KEY (Afghanistan OR Benin OR {Burkina Faso} OR Burundi OR {Central African Republic} OR Chad OR Congo OR Zaire OR Eritrea OR Ethiopia OR Gambia OR Guinea OR Guinea-Bissau OR Haiti OR {North Korea} OR Liberia OR Madagascar OR Malawi OR Mali OR Mozambique OR Nepal OR Niger OR Rwanda OR {Sierra Leone} OR Somalia OR {South Sudan} OR Sudan OR Syria OR Tajikistan OR Tanzania OR Togo OR Uganda OR Yemen OR Angola OR Bangladesh OR Bhutan OR Bolivia OR {Cabo Verde} OR {Cape Verde} OR Cambodia OR Cameroon OR Comoros OR Congo OR {Cote d'Ivoire} OR Djibouti OR Egypt OR {El Salvador} OR Swaziland OR Eswatini OR Ghana OR Honduras OR India OR Indonesia OR Kenya OR Kiribati OR Kyrgyzstan OR {Kyrgyz Republic} OR Laos OR Lesotho OR Mauritania OR Micronesia OR Moldova OR Mongolia OR Morocco OR Myanmar OR Burma OR Nicaragua OR Nigeria OR Pakistan OR {Papua New Guinea} OR Philippines OR {Sao Tome and Principe} OR Senegal OR Melanesia OR {Solomon Islands} OR Timor-Leste OR Tunisia OR Ukraine OR Uzbekistan OR Vanuatu OR Vietnam OR {Middle East} OR {Gaza Strip} OR Zambia OR Zimbabwe OR Albania OR Algeria OR {American Samoa} OR Argentina OR Armenia OR Azerbaijan OR Belarus OR Belize OR {Bosnia and Herzegovina} OR Yugoslavia OR Botswana OR Brazil OR Bulgaria OR China OR Colombia

OR {Costa Rica} OR Cuba OR Dominica OR {Dominican Republic} OR Ecuador OR {Equatorial Guinea} OR Fiji OR Gabon OR Georgia OR Grenada OR Guatemala OR Guyana OR Iran OR Iraq OR Jamaica OR Jordan OR Kazakhstan OR Kosovo OR Lebanon OR Libya OR Malaysia OR Maldives OR Micronesia OR {Marshall Islands} OR Mexico OR Montenegro OR Namibia OR {Republic of North Macedonia} OR Paraguay OR Peru OR Russia OR Samoa OR Serbia OR {South Africa} OR {Sri Lanka} OR {Saint Lucia} OR {St. Lucia} OR {St. Vincent and the Grenadines} OR {Saint Vincent and the Grenadines} OR Suriname OR Thailand OR Tonga OR Turkey OR Turkmenistan OR Tuvalu OR Venezuela)

S5 AND S6

Filters applied were also the same as original search strategies:

Subject Area Limit to Environmental Science

Keywords limit to "Chlorination", "Water Treatment", "Drinking Water", "Disinfection", "Chlorine", "Water Quality" (see the screenshots above)

Language Limit to English, Chinese, French and Spanish

<u>ProQuest Dissertations & Theses Global search</u>

S1 = ab(chlorinator OR chlorinate OR chlorination OR "chlorine dosing" OR "chlorine doses") OR ti(chlorinator OR chlorinate OR chlorination OR "chlorine dosing" OR "chlorine doses") OR diskw(chlorinator OR chlorinate OR chlorination OR "chlorine dosing" OR "chlorine doses")

S2 = ab("water quality" OR "drinking water" OR inline OR in-line OR "water treatment") OR ti("water quality" OR "drinking water" OR inline OR in-line OR "water treatment") OR diskw("water quality" OR "drinking water" OR inline OR in-line OR "water treatment")

S3 = S1 AND S2

S4 = ab(wastewater OR "waste water" OR sewage) OR ti(wastewater OR "waste water" OR sewage) OR diskw(wastewater OR "waste water" OR sewage)

S3 NOT S4

Index term (keyword) Include Drinking water, Water treatment, Water quality, Disinfection, Chlorination, Chlorine, Drinking water treatment, water treatment

Language Include English, French, Chinese (because Spanish is not found)

Flow Diagram

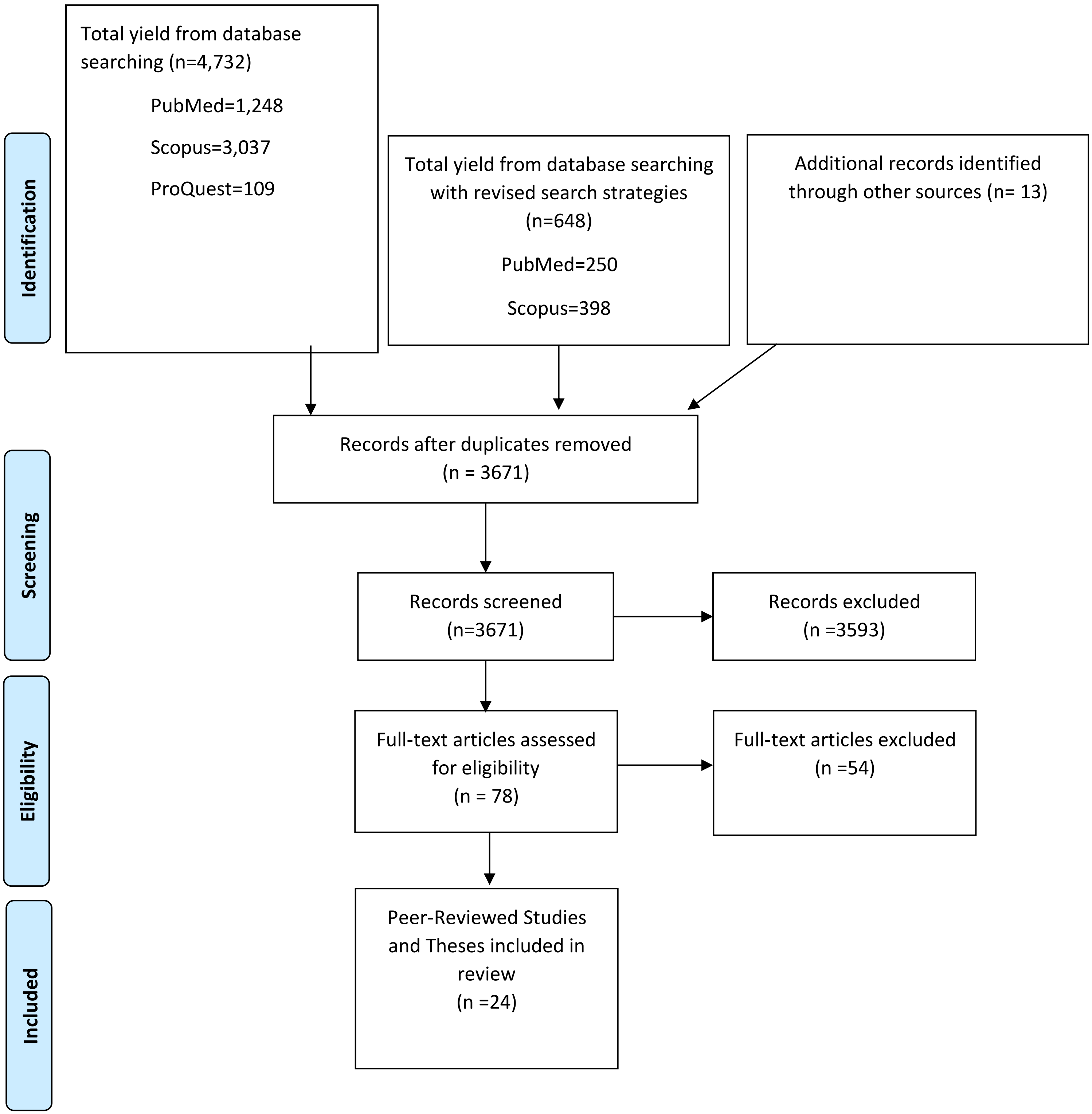

Figure S1. Flow diagram for literature search process including initial search, screening and inclusion steps.

2. NGO Survey
Determined to not be human subjects research by University of Iowa IRB on 03/17/2021

Survey Form available at: https://forms.gle/d1dCCJNFTMef1q527

Survey Questions:

1. Organization
2. Please name or describe the chlorinator(s) used by your organization. If you are uncertain how to describe the chlorinator feel free to upload an image at the end of this form.
3. Where does your organization implement chlorinators? (Please describe geographic location, community types, health care or school settings, etc)
4. Has your organization (or another organization) assessed the effectiveness of these chlorinators-if so, how? (please share a link to any publicly available evaluations or upload file at the end of this form)
5. Does your organization routinely monitor chlorinators you've implemented? If so, how?
6. Are there any other organizations using or testing chlorinators you recommend reaching out to? If so, please share a webpage or contact below.
7. If you are open to us contacting you for more information, please share an organization email address.
8. You can upload pictures, reports, or additional information you may want to share here.
